# Digital Technology Supporting the Remote Human-Dog Interaction: Scoping Review

**DOI:** 10.3390/ani13040699

**Published:** 2023-02-16

**Authors:** Liliana Rodríguez-Vizzuett, Ismael E. Espinosa-Curiel, Humberto Pérez-Espinosa

**Affiliations:** CICESE-UT3, Tepic 63155, Mexico

**Keywords:** animal computer interaction, dogs, human-dog interaction, interactive technology, remote communication

## Abstract

**Simple Summary:**

Due to the close affective and collaborative relationship between dogs and humans, in several situations there is a need to maintain communication when it is not possible to do it face to face. The objective of this review is to analyze the main aspects of current technologies that support remote communication between dogs and humans. Fifteen articles were selected which were conscientiously analyzed. The most widely used technologies to allow dogs to generate messages are wearable devices equipped with sensors. The most used technologies for dogs to receive messages are wearable devices equipped with vibrotactile actuators. Most of the proposals developed only include one-way communication, and those that include bidirectional communication uses videochats. All reported evaluations were pilot studies with positive feasibility results. The use of technology to support remote human-dog interaction is generating a lot of anticipation and excitement. However, there is still a long way to go in terms of technological developments, integration into the activities and context of dogs, support for new modalities of dog interaction, adaptation of technology to the canine traits and the determination of its efficacy.

**Abstract:**

For thousands of years, dogs have coexisted with humans and have been adopted as companion pets and working animals. The communication between humans and dogs has improved their coexistence and socialization; however, due to the nature of their activities, dogs and humans occasionally lose face-to-face contact. The purpose of this scoping review is to examine five essential aspects of current technology designed to support intentional communication between humans and dogs in scenarios where there is no face-to-face contact: (1) the technologies used, (2) the activity supported, (3) the interaction modality, (4) the evaluation procedures, and the results obtained, and (5) the main limitations. In addition, this article explores future directions for research and practice. The PRISMA-ScR (Preferred Reporting Items for Systematic Reviews and Meta-Analyses extension for Scoping Reviews) guidelines were followed when conducting the review. Scopus (Elsevier), Springer-Link, IEEE Xplorer, ACM Digital Library, and Science Direct were used as data sources to retrieve information from January 2010 to March 2022. The titles and abstracts were individually reviewed by the authors (L.R.-V., I.E.E.-C., and H.P.-E.), and the full articles were then examined before a final inclusion determination. 15 (3%) out of the 571 records that were obtained met the requirements for inclusion. The most used technologies for dogs are: (1) 71% of technologies focused on generating messages are wearable devices equipped with sensors (bite, tug, or gesture), (2) 60% of technologies focused on receiving messages are wearable devices equipped with vibrotactile actuators, and (3) 100% of technologies focused on bidirectional communication are videochats. 67% of the works are oriented to support search and assistance tasks. 80% of the works developed technology for one-way communication. 53% of the technologies have a haptic dog interaction modality, that is, there is an object that the dog must wear or manipulate in a certain way. All of the reported evaluations were pilot studies with positive feasibility results. Remote human-dog interaction technology holds significant promise and potential; however, more research is required to assess their usability and efficacy and to incorporate new technological developments.

## 1. Introduction

### 1.1. Background

Humans have the intuition that animals are intentional beings who know and feel and can communicate their intentions, knowledge, and feelings among themselves and humans [1]. Animals communicate through various modalities, such as visual, acoustic, semiochemical, or gestural behaviors [2]; therefore, the study of human-animal communication expands what can be considered language beyond grammar, words, and human language. In addition, a better understanding of how animals naturally communicate and the structure of their messages will significantly help develop more effective technological tools to assist human-animal communication. There are relevant precedents in the communication analysis between humans and different species of animals, for example, parrots [3], dolphins [4], and apes [5].

For thousands of years, humans and domestic dogs, also known as Canis familiaris, have formed close friendships and strong socialization bonds [6,7,8]. At the beginning of the domestication process (over 15,000 years), dogs were associated with human groups, and later the interaction between the two species intensified. Dogs began collaborating with humans in various activities such as hunting, herding, guarding, and pulling sleds [9,10,11]. Given the long-standing relationship between both species, dogs developed social-cognitive skills and abilities. Dogs can identify human social gestures and understand human communicative signals, especially, social signs [12], and human vocalizations [13]. Given those valuable capabilities, dogs were widely adopted as working animals to perform a wide range of support and assistance tasks [14,15]. Furthermore, as companion animals, dogs are able to positively affect psychologically and physiologically humans [16,17,18].

Humans and dogs occasionally lose face-to-face contact, for example, when a dog searches for a person in rural areas or when rescue dogs pass through extraordinarily narrow or difficult-to-reach locations for humans. In the case of companion animals, humans frequently leave their beloved dogs alone for long periods at home, which can lead to separation anxiety [19]. Supporting remote communication and interaction through current digital technologies opens up an exciting range of applications. These new technologies can enhance dogs’ abilities to perform companion tasks, search and assist, and improve their well-being [20]. However, the development of digital technology for animals poses many challenges. It is essential to discover the best communication and interaction technology that allows dogs to readily transmit messages to their owners or handlers, ideally with no or minimal training required, and that delivers messages from the human to the dog in a form that it can correctly understand.

Animal Computer Interaction (ACI) is a new branch of computer science that seeks to understand the interaction between animals and computer technology in contexts where animals live, are active, and socialize with members of their own or other species, including humans [21]. Relevant advances have been made in ACI to understand the aspects of usability and user experience critical in the design of animal-oriented interactive systems [22] and to develop interactive interface technologies for various species [23]. The design, development, and evaluation of interactive technologies that enable intentional communication between humans and dogs who do not have face-to-face contact is an exciting aspect of ACI. Various research efforts are being made to develop and evaluate technology in order to gain knowledge and facilitate remote human-dog interaction. Therefore, it’s crucial to comprehend how these solutions are created, put into practice, and assessed in terms of the following inquiries:

1. What digital technologies are employed to facilitate remote human-dog interaction?
2. What activities have been supported by remote human-dog interaction technology?
3. What interaction modalities have been used for remote human-dog interaction systems?
4. What are the types of evaluations applied to validate the technologies, and what are the primary outcomes assessed when validating remote human-dog interaction technology?
5. What are the reported limitations of technology employed for remote human-dog interaction?

### 1.2. Objective

To our knowledge, only one previous review addresses the use of technology for animal welfare. However, it is exclusively focused on smart computing and sensing technologies and for a broad range of species [24]. As a result, the goal of this study was to conduct a scoping review of scientific and technological advances in interactive technology for remote human-dog interaction. This review will help us better understand how this type of technology is designed, used, and evaluated by answering the five questions mentioned above. This article also analyzes the impact of digital interventions for remote human-dog interaction in various contexts and activities and explores future directions for research and practice.

## 2. Methods

To ensure that our review was conducted systematically and without bias, we conducted a scoping review using the PRISMA-ScR (Preferred Reporting Items for Systematic Reviews and Meta-Analyses extension for Scoping Reviews) methodology [25]. The study has not been registered in PROSPERO since it is not for human health.

### 2.1. Eligibility Criteria

The studies included in this scoping review were English-language research articles published in journals and conference proceedings between January 2010 and March 2022 that described (1) interactive digital technology with the goal of (2) supporting remote human-dog communication and interaction and included (3) an evaluation procedure. Thus, studies that (1) were not research articles, (2) were not written in English, (3) did not describe a digital interaction technology, (4) did not support remote human-dog or dog-human interaction, (5) did not include an evaluation procedure, (6) were literature reviews, and (7) were repeated were excluded.

#### 2.1.1. Information Sources

The databases used for this review were: Scopus, SpringerLink, IEEE Xplorer, ACM Digital Library, and Science Direct. These five databases were chosen because they are recognized as reliable sources of high-quality publications from computer science, technology, and engineering. The search also took into account some hand-searched papers that were cited in the articles that were retrieved.

#### 2.1.2. Search

The specific syntax of the queries varied depending on the database. However, the concepts of (1) digital interactive technology and (2) human-dog communication and interaction were always expressed using the same words. The following words were included in the query: (“dog”) AND (“assistance” OR “service” OR “search and rescue” OR “working” OR “companion”) AND (“technology” OR “wearable” OR “computer” OR “system” OR “platform”) AND (“interaction” OR “communication”).

#### 2.1.3. Study Selection

The screening process was carried out in stages. The titles and abstracts were initially screened by the three authors (LRV, IEEC, and HPE). The full texts of the selected articles by the three researchers were examined in a subsequent stage before final inclusion. When numerous publications were published for the same study or application, it was reviewed to see if there were any major differences in the evaluation, such as if it was evaluated with a different population or other variables. The data were independently examined and extracted by the reviewers, and any discrepancies were settled through discussion until an agreement was reached.

#### 2.1.4. Data Charting and Result Synthesis

The review included all studies that met the inclusion criteria, and the data extracted were those that allowed for the answers to the five questions listed in Section 1: (1) the type of technology (sensing gesture sensors, touchscreens, objects with capacitive sensors) aimed to support the remote human-dog interaction, (2) the type of activity supported (search-and-rescue, assistance, companion, or general purpose), (3) the dog interaction modality (haptic, sound, video, vibrotactile), (4) the reported findings in terms of the various outcomes related to remote human-dog interaction, and (5) the reported limitation of the technology. To extract and summarize the above data, the authors created, calibrated, and used a template with various sections. We proceed to describe the major findings that emerged from the studies.

## 3. Results

### 3.1. Overview

**Figure 1:**
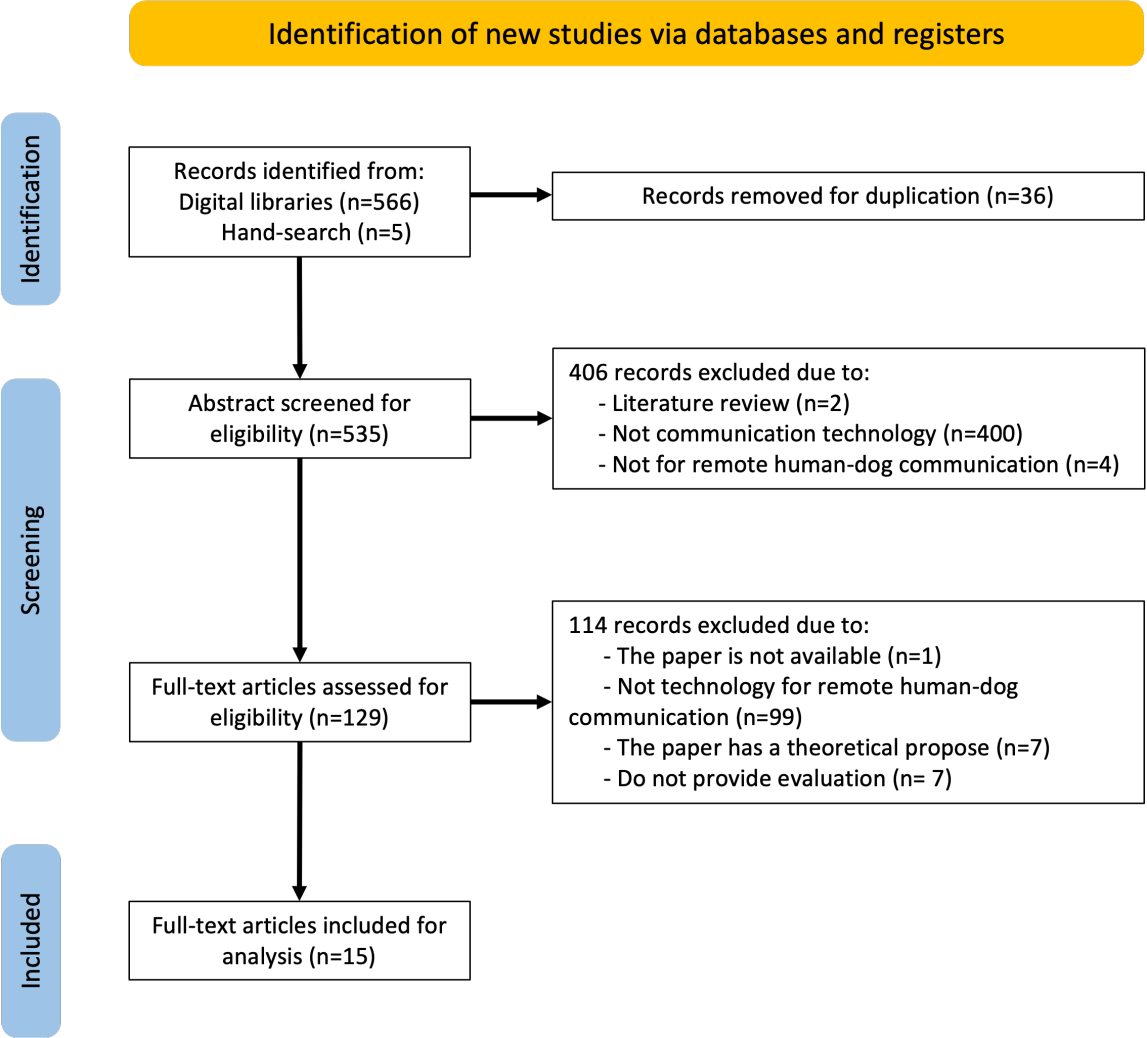
Flow diagram of the study selection.

The search resulted in the identification of 571 records. 99.1% (566/571) of the records were obtained from the five digital libraries, with an additional 0.9 percent (5/571) obtained through hand searching. After removing all duplicated records, 535 papers were screened for eligibility in the first stage. Based on the exclusion criteria, 75.9% (406/535) of the records were discarded after reading the titles and abstracts. After reviewing the full text of 129 articles, 94.1 % (112/129) were excluded. As a result, 15 studies were chosen for further examination. Figure 1 shows the flow diagram for the several stages of the review. Table 1 summarizes the overall findings. Table 2 presents, in chronological order, all the articles that were selected, analyzed, and summarized. The information in this table is the paper author, year, technology, main functionalities, addressed activities, communication direction, dog interaction modality, and evaluation. Next, we summarize the main findings to respond to the research questions.

### 3.2. Digital Technologies Implemented

According to our analysis, the digital technology implemented can be divided into three groups: technology for dogs, technology for humans, and technology for processing and interconnection.

#### 3.2.1. Technologies for Dogs

Dog technology can be divided into three categories: to send messages to humans, to receive messages from humans, or to send and receive messages. The technology to send messages to humans is mainly based on wearable devices with sensors [29,30,32,33], pulling sensor installed on a wall [31], and touchscreens [35,38]. To receive messages from humans, the technology is mainly based on vibrotactile actuators [36,37,39] and audio playback [28], or both, vibration and sound [26]. The technology to generate and receive messages is based on videochats [27,34,40]. Concerning wearable devices to generate messages by the dogs, some works [29,33] used harnesses with different sensors activated by bite, tug, and nose gestures. They used force-sensitive resistors to implement bite sensors, an ultrasonic range finder that detects nose movement at 3 cm. The tug sensor was made into an elastic band with a stretchable rubber variable resistor. Other wearable devices were developed [30,32] in order to identify head and body gestures, respectively. These systems obtain data from a collar that includes inertial sensors to detect gestures paired with predetermined behaviors. Other works [31] developed a detaching component that the dog could pull off to trigger a medical alarm. A couple of works [35,38] explored how to obtain dogs’ input with a touchscreen interface and the difficulties they have when interacting with this kind of device. Videochats allow bidirectional communication where dogs and humans can generate and receive messages synchronously. The DogPhone hardware prototype [40] includes an orientation sensor that combines an accelerometer, magnetometer, and gyroscope to detect movement, interpret the dog input, and start a phone call. In [27,34], a pet video chat system was designed using Skype’s audio-video connection with remote interaction features.

#### 3.2.2. Technologies for Humans

Mobile applications are the most common device to send or receive messages from dogs. In [28] used a mobile application to send spoken commands to the wearable device. In [36,37] used a mobile application to send vibrotactile commands to the wearable device of search-and-rescue dogs. In [30] used a mobile application that receives sensor readings via a Bluetooth connection and plays synthesized speech messages of the gesture being performed. A corresponding message is communicated if the collar is out of range or more than five samples were skipped. In another work [33], the authors developed an application that receives notifications when the dog bites the sensor. The application also shows the dog’s location concerning the handler, a compass, and general wind direction. In the system developed by Golan et al. [39], a vibrator in the dog’s harness was activated by a handheld transmitter (remote control).

#### 3.2.3. Technologies for Processing and Interconnection

For processing and interconnection, most studies used an all-in-one development board. In [33] they used a central hub with a Bluetooth radio to broadcast sensor activation feedback tones to the dog and send alerts to a cell phone. An Arduino board that activates the appropriate vibrators and interconnects via WiFi to a mobile application was used in Morrison et al. [36]. The Adafruit Feather Huzzah ESP8266 board, which included a Built-in WiFi 802.11 b/g/n was used in [40]. In [37], the authors used the Intel Next Unit of Computing (NUC) KIT NUC5i3RYH to control the prompts displayed on the screen, manage the interactions, and upload the data to the server. In the case of human technology, all the processing and interconnection were made in the smartphone. In [28], two prototypes were developed; the prototype is a harness equipped with speakers connected to a smartphone that is attached to the harness. The smartphone is connected to two amplified speakers attached to the harness under the dog’s ears. Voice commands can be activated remotely with a second smartphone. The two smartphones communicate using the Direct WiFi standard that allows a distance of 50 m between the two smartphones without much delay to reproduce the sounds. The second prototype was for detecting dog activity. Two smartphones were used for this prototype, adding the 3-axis accelerometer and 3-axis gyroscope. The smartphone is placed on the dog’s back, and the sensors with a specific location so that the sound can be reproduced correctly.

### 3.3. Activities Intended to Support

According to our analysis, the main activities supported by remote interaction technology can be grouped into assistance, search-and-rescue, companion, and general-purpose activities.

#### 3.3.1. Assistance and Service Activities

Assistance dogs have become part of the daily life of many people with conditions that limit them from carrying out their daily activities. In terms of assistance activities, the works have addressed technology allowing assistance dogs to alert in case of events that require attention or in an emergency and ask for help on behalf of their owners [31,32,35], and generate medical alerts by operating emergency notification systems [37,38].

#### 3.3.2. Seeking, Locating, and Rescuing Activities

Dogs that assist in seeking, locating, and rescuing activities are called search-and-rescue dogs [41]. We found that remote interaction technology has been designed for search and rescue and hunting dogs. Both tasks have in common the use of their powerful sensory abilities, mainly olfactory, to locate a target. Search and rescue (SAR) dogs are trained to locate people in extreme situations, in terrain that is often difficult for humans to access: in the snow, in the open air, in the mountains or at sea, and after earthquakes and other catastrophes that can generate large amounts of rubble. Concerning SAR dogs, we identified two studies. The first study is to alert the handler when a SAR dog finds something interesting [33]. This system sends a signal via cell phone to the handler’s smartphone, including GPS data and activation information. As the SAR dog moves, a trajectory is drawn on the map showing where the dog has searched. In [26], the authors developed a system that tracks a canine’s position, motion behavior, and orientation. It also supports the remote actuation of tone and vibration commands and reports commands in real-time alongside sensor data. For the case of hunting dogs, in [36] created a vibrotactile vest (VTV) to give commands to execute the tasks for which they were trained. Valentin et al. [30] proposed a system including a collar and an app for dangerous tasks such as search and rescue or explosive detection. The collar identifies specific movements of the dog’s head, and the app receives messages about the movements detected. Golan et al. [39] also tackled complicated scenarios humans cannot do alone, such as detecting explosives or searching narrow spaces. They implemented a vest with four embedded vibration motors. The vest applies vibrotactile cues to the dog that wears it, and the dog is trained to associate the cues with useful commands.

#### 3.3.3. Companion Activities

Companion dogs live in their owners’ homes and may learn to perform specific tasks. The research in this category is motivated by the bond between humans and domesticated dogs and the need to stay connected from distant places. The following three studies were identified in this category. In [27], investigate the potential of interactive cameras for dogs throughout a pet video chat system to augment Skype’s audio and video connection with remote interaction features. The work by Rossi et. al. [34] aims to show the ability of a canine to provide verbal cues given through a video chat. The authors used the software Skype and an automatic kibble dispenser to improve the bond between domestic dogs and their owners through remote audiovisual interaction. Communication was bidirectional from the human to the dog and vice-versa. The owner communicated with the dog to give directions, and the dog could emit a vocalization or another signal in response. A more recent studio explores the creation of a video call device to allow a dog to initiate a video call with its owner [40]. In this case, the dog has control over this home communication device. The authors chose a tennis ball as the interface to initiate the call because the behavior of biting such an object already had a prior meaning and use for the dog-human relationship.

#### 3.3.4. General-Purpose Activities

These works have developed remote interaction technology without focusing on a specific activity. In this category, one study focused on a technology that enables dogs to communicate events through several interfaces that detects head movements, bites and tugs [29]. In addition, other study focused on technology that allows humans to provide audio cues and commands [28] to dogs in several scenarios or activities. The authors argue that this technology may benefit activities such as training and communication with deaf dogs and training by handlers with speech impairments.

### 3.4. Interaction Modalities

The interaction modalities are the means that allow the user (dog or human) to communicate with the computer system, that is, they allow it to generate the input or receive the output. According to our analysis, the interaction modalities can be divided between those that are for dogs and humans.

#### 3.4.1. Interaction Modalities for Dogs

The primary interaction modalities for dogs are haptic, sound, and audio/video. Dogs can bit, tug, and make nose gestures [29,33], nose touching [35,38], head and body movements [30,32], and pulling a rope to generate messages [31]. Three works explore the interaction through audio/video [27,34,40]. Most works that send messages to the dog use vibrotactile devices [36,37,39]. In addition, two works used sound to provide a message to dogs. The first is speech prompts [28], and the second uses different tones that correspond to the commands forward, stop, and recall [26].

#### 3.4.2. Interaction Modalities for Humans

Several of the identified works only focus on the design of technology for dogs and do not provide an interface for humans [26,29,31,35,38]. In the works that include interaction technology for humans, most are touch interfaces into mobile apps to send [28,37] or receive messages [30,32,33,36], and wireless remote devices to send commands [39]. Finally, three works use an audio/video interface to support that humans interact with dogs [27,34,40].

### 3.5. Evaluation Procedure and Reported Results

All the analyzed studies conducted exploratory studies to evaluate the feasibility of the proposed technology. In particular, most studies focus on assessing dogs’ capacities to use the technology and the time required to train them to use it. The evaluations were conducted in pilot studies with 1 to 12 dogs; nevertheless, most were conducted with four dogs. Next, we analyze the evaluation studies and the reported results.

#### 3.5.1. Evaluation of Technologies to Support Dogs Sending Messages

We identified that most of the studies that propose technology that enables dogs to send messages focused on evaluating the capacities of dogs to use the technology and the time required to train them to use it. The evaluation of vests and collars focused on validating if dogs can reliably activate the sensor mounted on them to interact with their handlers.

Variables such as training time, dog accuracy, sensor accuracy, sensor range, and overall success were measured [29,32]. The authors were able to verify that wearable electronics mounted on harness can be reliably activated by dogs to interact with their handlers. However, the sensors must be more compact, durable, and power-efficient. In addition, requirements related to durability, visibility, and connectivity of the vest and the mobile application’s mapping, iconography, and annotation were identified [33]. They showed the viability of the proposed technology by evaluating it with feedback from expert trainers. Also, it was evaluated if dogs can use a wearable device such as a collar or vest that detects a set of gestures for dogs to communicate with handlers [30]. Their findings demonstrated the kinds of gestures that can help working dogs communicate important information to trainers and the significance of taking into account the devices the dog is currently wearing, such as a leash, harness, or existing collar, when choosing gestures.

Similarly, the evaluation of touchscreen interfaces focused on validating their usability and precision. In [35], the authors evaluated the usability of a touchscreen interface by training five dogs on the take-off tapping task. In previous work, the same author [42] had evaluated their touchscreen-based system by counting the number of touches of the dogs vs. time, the time of the dogs vs. distance from the edge of the circle, and the time of the dogs vs. difficulty index. They highlighted a number of best practices, including the use of infrared touchscreens with non-projection monitors as the background, the need that tapping targets be at least 3.5" long, and the fact that shape is the most efficient mode of instruction for touchscreen interactions. Byrne et al. [38] assessed the feasibility of using a touch screen as a real-time medical alert system. They showed how dogs may be taught to use their noses to press a sequence of touchscreen symbols to transmit a medical alert. Even dogs with no prior touchscreen training can learn a complicated alert behavior chain in less than a week with just daily training sessions of five minutes.

Similarly, a medical alert system activated by pulling a rope was evaluated by Robinson et al. [31]. The results of this evaluation were to provide a series of recommendations on user-centered design for assistance dogs and humans to develop a system that would allow assistance dogs to call for help remotely.

#### 3.5.2. Evaluation of Technologies to Support Dogs Receiving Messages

The studies that propose technology for supporting dogs receiving remote messages focused on evaluating the capacities of dogs to interpret these messages and the time required to train them to use them. In [28], the authors verified that dogs could obey a recorded vocal command of the owner’s voice when it is not in visual contact with their owner. In addition, several works evaluated how well dogs interpret orders remotely sent and played by vibrotactile actuators. In [36] the authors measured interaction variables; whether the dog was treated or praised, the lack or type of mark the dog made, whether the dog looked away, looked at the facilitator, followed the facilitator’s hand with its head, or walked towards the facilitator, and the number of times it responded correctly to the command. They emphasize the significance of accurately identifying previous training methods and planning modified trial settings in advance to accommodate each dog’s unique learning experience. In the evaluation reported in [37], the authors measured the accuracy of the dog’s response to a series of stimuli; these responses are divided into three variables: Deletions (D), Substitutions (S), and Insertions (I). They demonstrated that dogs could be trained to respond to vibrotactile cues. Moreover, in [39], the evaluation was carried out with a dog trained to associate four different types of vibrations with other commands to assess the number of successfully performed orders. They proved that instructing dogs to carry out numerous activities using vibrotactile cues was quite effective. The test subjects responded well to a single haptic command, coming close to matching the vocal command sensitivity. In [26], the authors measured the success rate for simple and more complicated multi-point paths where dogs had to leave the point of origin, go to a waypoint, stop, and then return to the end of the head. A “success” means that the canine came close enough to the destination waypoint and stopped when commanded. A “failure” indicates that the dog could not be commanded to arrive at the waypoint. They demonstrated how the sensor data may be used to recognize when a dog assumes a different stance in addition to guiding the dog to a predefined place.

#### 3.5.3. Technologies for Bidirectional Communication

The studies that propose bidirectional communication technology are mainly audiovisual systems used by pet dogs. In [34], it is shown that the dog responds to commands given from a distance and that video call interactions can benefit the dog. To evaluate the use of DogPhone [40], the authors employed HCI’s established mixed-method approach of combining a diary study and recommended interpretations from the human side with quantitative interaction data from the DogPhone interactions. They examine how interactions should be managed, how to measure interactions, how dog devices are created through prototyping, and what these things entail for dogs. In [27], the effectiveness of the pet video chat was tested. The results are encouraging for pet video chat systems that allow owners to see and interact with their pets while away. They demonstrated how it is essential to be able to see the animal in order to properly promote interaction.

### 3.6. Limitations of Remote Human-Dog Communication Technology

Although significant advances have been made with current technologies, the studies identified the following limitations that should be considered. Jackson et al. [29] identified that sensors need to be smaller, robust, and less power-consuming to adapt to the characteristic of dogs. In addition, the breed and dog body types affect the effectiveness of the technology. Coat density, body shape, and fat/muscle distribution could affect the fidelity of the message conveyed. In addition, differences in cognition and experience can also be a problem. These issues also affect the design and positioning of the sensing devices [30]. In addition, other limitations are related to the balance between canine and human requirements, an issue that must be considered during the design process [31]. Finally, the evaluation is a limitation since more research is necessary to test their results’ validity, reliability, reproducibility, and generalization. Future efforts should focus on trying the technologies in a more significant number of dogs of different breeds, ages, and training histories [39].

## 4. Discussion

### 4.1. Principal Findings

Considering the rising interest in building digital technology to enable remote human-dog communication, future research should highlight critical elements that remote human-dog communication designers should consider. This scoping study helps by identifying and summarizing the description of five key features that characterize how these devices are currently constructed and the intervention results provided. The results discussion is organized around the primary objectives addressed in this scoping review.

#### 4.1.1. Digital Technologies Implemented

The results obtained from this literature review indicate that the development of technology to support communication between dogs and humans is still incipient. It has been possible to validate suitable interfaces for dogs to send messages (touch screens, devices activated by biting, pulling, and gestures) and receive messages (vibrating vests, speakers). Wearable devices like harnesses, collars, and modified toys (balls, rope toys) have been studied extensively. The prototypes include a variety of sensors and actuators for two-way communication.

Undoubtedly, the results achieved are valuable and relevant for the design of dog-computer interfaces. However, it is clear that these technological proposals still need to mature and be evaluated more extensively, as well as testing other means of communication, taking advantage of dogs differentiating characteristics. Therefore, there is a need to investigate new sensors and actuators, for vocal or olfactory interaction, for example. Most of the revised works focused only on one-way communication technology (dog-human or human-dog), and few works focused on bidirectional communication, all of them videochats. Further research is needed to integrate the advance of one-way communication technology to develop bidirectional communication systems.

Information technologies such as the Internet of Things, augmented and virtual reality, big data, 3D printing, and artificial intelligence have not been exploited when implementing prototypes for remote human-dog interaction. In the coming years, these technologies have the potential to enable significant advances in remote human-dog interaction. In order to scale current technological solutions, it is necessary to integrate these trending technologies into more robust communication platforms taking advantage of their benefits. Surprisingly, advances in technology for interaction with dogs are not as outstanding as expected, given the very close human-dog relationship. It is perceived that the area of animal-computer interaction is in the early stages of growth, specifically in dog-computer interaction, in which it is beginning to take inertia thanks to the push of a few research groups and the financing of projects around the world. In later stages, it is expected that synergy will be generated between the different groups to create shared resources and tools, leading to a more accelerated advancement of the area.

#### 4.1.2. Activities Supported by Remote Human-Dog Interaction Technology

Significant efforts have been made to implement technology to support remote interaction in assistance, search and rescue, hunting, companionship, or general-purpose dog activities. However, according to the taxonomy of assistance animals proposed by Parenti et al. [41], many activities could benefit from human-dog remote interaction technology. For example, guide, autism, herding, emotional support, mobility assistance, and patrol. Applying this technology to these activities could even revolutionize how dogs currently perform these activities. Additionally, while the identified technologies were created to support specific activities, they can be adapted to new situations and activities with minor changes. However, more research is needed to determine the viability and implications of these actions.

#### 4.1.3. Interaction Modalities

Dogs’ key interaction modalities to create signals include nose touch, bite, pull, tug, body, head and nose movements, and audio/video. Furthermore, the key interaction modes for receiving signals are vibrotactile and sound.

There are interaction modalities that have not yet been investigated and used in both circumstances, taking into consideration the order of significance of dogs’ senses (smell, hearing, vision, touch, and taste) [28] and dogs’ communication methods (e.g., touch, vocalizations, and movements). For instance, a vocal interface for dogs to create messages, assuming that the dog expresses itself through vocalizations. Another example is a system that reads dog body motions and converts them into messages. Another option is to use the dog’s sense of smell to create an odor-based interface for communicating with them. Concerning interaction technology for humans, most prototypes generate messages through touch interfaces implemented into mobile devices. Mobile apps are the most common interface for receiving visual or audible messages. Similarly, human vocal interfaces could send direct audio messages to dogs.

It is important to mention that in all the studies safe technologies were proposed in terms of canine well-being. The devices that could be less comfortable for dogs are harnesses and vests since due to the electronics with which they are equipped they can be heavy and generate a little heat. However, the signals sent by these devices to dogs are harmless as they are mainly mild vibrations.

#### 4.1.4. Evaluation and Results

Some human-oriented interface design techniques have been used in research efforts with dogs. However, a methodological adaptation and specialization phase are still required to create effective human-dog interaction interfaces. On the other hand, most of the current validations of human-dog interaction interfaces are inconclusive because they were conducted with a small number of test subjects or prototypes in the early development stages.

#### 4.1.5. Technology Limitations

The constraints noted in the papers under consideration are connected to the difficulty of generalizing system design advances. Depending on the breed, dogs have a variety of physical and behavioral characteristics. It is worth noting that the validations were only done with a few dogs (between one and four). As a result, there is insufficient data to draw definite judgments. Several challenges developing interspecies communication technologies can be addressed. Canines that function as therapy, assistance, skilled companion, and service among others [43], have piqued the curiosity of the scientific community.

### 4.2. Gaps in the Research

The following areas of opportunity and research requirements to support the development of this type of technology came from the findings of this review.

- Creation of cutting-edge new technology. Wearable technologies, touchscreens, video and audio interfaces, specialized network systems, and artificial intelligence advances must be integrated to enable future developments that allow humans and canines to execute sophisticated remote socializing and collaboration tasks more naturally.
- Integrate new cross-application research. There is a need to build technology that can be utilized easily and effectively in diverse environments or for different activities.
- Create new interaction modalities. It is necessary to build and create new dog-computer interfaces and multimodal communication systems that consider the whole range of a dog’s senses and interaction methods.
- Develop dog-centered technology. There is a need to shift the technology design paradigm in favor of one focused on the characteristics of dogs. For instance, create small and low-power devices considering the dogs’ breed, size, and body type.

## 5. Limitations

This review raises critical issues that should be taken into consideration when interpreting the results of this review. One of these disadvantages is that only studies that provided information on an evaluation process were considered. Some advanced technology (electronic devices with machine learning or other artificial intelligence methods) was not considered due to a lack of testing. Furthermore, most studies do not include a medium-term evaluation of intervention efficacy in their research design to confirm the generalizability of the developed technology. Another limitation is the small number of included studies that were examined. Due to the possibility that some other pertinent studies were missed during the search, this study’s database consideration is also constrained. If too many databases are used, the search may be predisposed to excessive, unjustified duplicates of the searched results, even though the five databases that were searched may overlap with other databases. However, we considered that this study makes a significant contribution because it shows the state of the art in this area of technological research and development. In addition, this study also highlights the need for more research in this area because there have only been a few publications in this particular field.

## 6. Conclusions

The results highlight digital technology’s significant promise and potential to support remote human-dog interaction. Wearable technology for dogs and mobile apps for humans was the most extensively studied technologies for remote human-dog interaction. Most technologies were created to aid dogs in their assistance activities, and the most commonly reported interaction mode for dogs was haptic. Most of the reported evaluation protocols are pilot studies with fewer dogs that reported positive results regarding the feasibility of the technology focusing on assessing dogs’ capacities to use the technology and the time required to train them. The use of technology to support remote human-dog interaction generates much expectation and excitement. However, there remains a long way to go regarding technological developments, integration into the activities and context of dogs, supporting new dogs’ interaction modalities, adapting the technology to the dog’s characteristics, and establishing effectiveness.

## Figures and Tables

**Table 1:** Overview of the main characteristics of the reviewed technology (n = 15).

Characteristic	Studies, n (%)
Interfaces for dogs	
To generate messages	
Touchscreen	2(13)
Bite sensor	1(7)
Tug sensor	1(7)
Gesture sensor, bite sensor, tug sensor	1(10)
Gesture sensor	2(13)
To receive messages	
Vibrotactile	3(20)
Audio	1(7)
Audio and vibrotactile	1(7)
To generate and receive messages	
Videochat	2(13)
Videochat and bite sensor	1(7)
Interfaces for humans	
To generate messages	
Mobile application	3(20)
Handheld transmitter	1(7)
To receive messages	
Mobile applications	3(20)
To generate and receive messages	
Videochat	3(20)
Do not show human interface	5(15)
Activities addressed	
Search-and-rescue	5(33)
Assistance	5(33)
Companion	3(20)
General purpose	2(14)
Interaction modalities for dogs	
Haptic	8(53)
Haptic and sound	1(7)
Video and sound	2(13)
Sound	1(7)
Wearable	2(13)
Video, sound and haptic	1(7)
Evaluation protocol	
Pilot study with dogs	14(93)
Pilot study with trainers	1(7)

**Table 2:** Summary of the main characteristics of the reviewed technologies (n = 15).

Technology	Year	Main Functionalities	Dog Activities Addressed	Message Direction	Dog Interaction Modality	Evaluation Protocol	Dog Training Sessions	Main Reported Results
1. Harness with sensors and actuators [26]	2011	Vest that allows handlers to command and track a trained canine in real-time using a sensor suite and a tone generator.	Search-and-rescue	Human to Dog	Haptic and sound-Vibrations and audio commands	Pilot test-1 dog	The dog had previous field/hunt trials and explosive detection training. The dog was trained to respond to tones and vibrations, but no training session details are provided.	The system can use data from the canine’s sensors to provide audio and vibration commands and control signals to autonomously guide the dog to destinations and send information to the handler.
2. PC with Skype [27]	2012	Video chat system that augments a Skype audio-video connection with remote interaction features.	Companion	Bidirectional	Video and sound	Pilot test-10 dogs	Authors do not mention the dog’s previous training. The dogs were trained to interact with the screen but no training session details are provided.	They demonstrated the potential of pet-based videochat systems that enable owners to watch their pets while not at home and communicate with them via audio and visuals, promoting animal engagement.
3. Harness with speakers [28]	2013	Vest allowing to command the dog through an embedded voice and recognizes some activities of the dog: walk, seat, run, lying.	General	Human to Dog	Hearing commands	Pilot test-1 dog	The participant dog was already familiar with the basic vocal commands. The authors do not specify a training period.	They demonstrated that dogs obey recorded vocal commands. Furthermore, using their system, the handler can remotely monitor the dog’s activity.
4. Harness with sensors [29]	2015	Five different haptic sensors that dogs could activate based on natural dog behaviors such as biting, tugging, and nose touches.	General	Dog to Human	Haptic-Bit, tug, and nose gestures	Pilot test-3 dogs	The dogs had previous assistance training. No more than four sessions per day, with a 30-min break in between, were held for sensor-specific training, and each session lasted no longer than 15 min.	It is feasible to design wearables that canine handlers can dependably activate. 100% of the commands transmitted through capacitive and pneumatic sensors resulted in successful activations for all eight dogs.
5. Collar with sensors [30]	2015	Collar that detects gestures using a motion sensor. Each gesture is paired with a predetermined message that is voiced to the handler by a smartphone.	Search-and-rescue	Dog to Human	Gestures recognition	Pilot test-3 dogs	The dogs had previous training in alert, assistance, and police tasks. The gesture training occurred in at most four 30-min long sessions for each dog.	The authors demonstrated that working dogs could use gestures (spin, twirl, right sequence, left sequence) to communicate with humans. The results when evaluating the gestures recognition collar were not totally satisfactory since important aspects to improve in the design were detected.
6. Trigger that activates an alarm upon detachment [31]	2015	Alarm system that enables assistance dogs to call for help using a detaching component that the dog could pull off to trigger the alarm.	Assistance	Dog to Human	Haptic-Pulling rope	Pilot test-4 dogs	All dogs in this study already knew how to ’tug’ and retrieve on command. The dogs were trained to use the mechanism but no training session details are provided.	The main contribution was the set of lessons learned from the particular design application and design process that included the dogs, their owners, and trainers in the process.
7. Harness with movement sensors [32]	2016	Collar that senses gestures using an inertial measurement unit and relays specific alerts to a smartphone application.	Assistance	Dog to Human	Gestures recognition	Pilot test-2 dogs	Only one dog had previous experience with gestures. Each training session lasted at most 10 min. The learning time for each gesture varied depending on the dog’s prior training experience but did not exceed 15 training sessions per gesture.	Dogs were successfully trained to perform gestures. However, the sensing harness was not evaluated. After training, dogs could continue to make the signals without the harness, but less precisely. Dogs still recall the gesture when prompted with a verbal and physical cue over three months.
8. Harness with bite sensor [33]	2016	Wearable bite sensor for search-and-rescue dogs that communicates with their handler via a mobile application.	Search-and-rescue	Dog to Human	Haptic- bite	Pilot test-3 trainers	The dogs had previous training in search and rescue. A dog was trained to use the vest, but no training session details are provided.	Three K9-Search and Rescue experts evaluated the system. They recommended improvements to the vest for durability, visibility, and connectivity to the handler; and improvements to the app regarding mapping, iconography, and annotation.
9. PC with Skype [34]	2016	Video call interactions with the dog and a treat dispenser triggered to release food from a distant location.	Companion	Bidirectional	Video and sound	Pilot test-1 dog	The dog was trained to correctly respond to verbal cues requested through Skype, but no training session details are provided.	The video capability not only gives the owner the option to check on their pet frequently to ensure that it is safe, but it also allows them to engage in meaningful communication with the pet.
10. Touchscreen [35]	2016	Touchscreen interfaces usable for assistance dogs in the home. Validation of interaction techniques such as lift-off selection and sliding gestural motions.	Assistance	Dog to Human	Haptic-Nose touching	Pilot test-5 dogs	The dogs were trained in a 15–20 min sessions with at least 30 min rest between each training session.	The most effective technology for canine interaction is infrared touchscreens with backing projection monitors. The most efficient training technique for touchscreen interactions involving tapping is shaping. Luring can be used successfully to train sliding/gestural interactions at first, but it should be swiftly replaced with shaping.
11. Harness with vibrator [36]	2016	Harness to provide vibrotactile commands to dogs, working with variable-intensity vibrating motors mounted to a modified hug shirt.	Search-and-rescue	Human to Dog	Haptic-vibrations	Pilot test-4 dogs	The dogs had previous hunting, track, and obedience training. A dog was trained to correctly respond to vibrotactile cues, but no training session details are provided.	The authors tested the design of the vest and vibrating actuators. They concluded that it is crucial to correctly identify previous training methods and prepare modified experimental settings that consider each dog’s learning experience.
12. Harness with vibrator [37]	2017	Vest with vibration actuators at different points on the dog body that is evaluated measuring the working dog’s ability to perform distinct tasks.	Assistance	Dog to Human	Haptic- vibrations in shoulders and bite	Pilot test-11 dogs	The dogs had diverse previous training. Training sessions were no more than fifteen minutes long. Each dog had no more than four training sessions per day, with at least thirty minutes between them. Training sessions were conducted until the dog mastered the haptic cue.	They demonstrated that canines can be taught to react to haptic stimuli. Over 93% of haptic cues resulted in the dog reporting perceiving the cue, for the highest power level of vibration. Not surprisingly, the lower power levels resulted in lower Dog Response Rates, with the lowest power level under 57%.
13. Touchscreen [38]	2018	Touchscreens mounted in the home triggered by the dog interaction to alert in emergencies.	Assistance	Dog to Human	Haptic-Nose touch	Pilot test-3 dogs	The dogs had diverse previous training and diverse experience with touchscreens. Dogs require less than 40 min of total training time, spread out over less than a week.	Dogs can be taught to use their noses to press a sequence of touchscreen icons to signal a medical emergency in less than a week with just five-minute training sessions each day. Dogs can locate the touchscreen from different rooms and only activate the touchscreen only when given the training cue.
14. Harness with vibrator [39]	2019	Harness embedded with vibration motors associating four different types of vibrations with different commands.	Search-and-rescue	Human to Dog	Haptic-vibrations in shoulders	Pilot test-1 dog	The dogs had never received any formal training. The dog was trained to correctly respond to vibrotactile cues, but no training session details are provided.	Vibrotactile indications successfully directed dogs to carry out several tasks (turn around, lie down, approach handler, walk backward). Dogs responded well to a single haptic command, matching their vocal command sensitivity.
15. PC with Skype and bite sensor [40]	2021	Video call device to allow a dog to remotely call their human, giving the animal control and agency over technology in their home.	Companion	Bidiectional	Haptic, video and sound	Pilot test-1 dog	The dog had no previous professional training but had previous technology experience with screen devices and motion and facial trackers. The trainer performs five use demonstration actions to teach the dog how to use the ball to call.	Thanks to the system, dogs could video call their human whenever and wherever they wanted. The experimental design provided knowledge on how to create Internet of Things systems using canines. Through prototyping, dogs were incorporated into the early stages of design.

## Data Availability

Not applicable.
